# Prospect on Ionomic Signatures for the Classification of Grapevine Berries According to Their Geographical Origin

**DOI:** 10.3389/fpls.2017.00640

**Published:** 2017-04-24

**Authors:** Youry Pii, Anita Zamboni, Silvia Dal Santo, Mario Pezzotti, Zeno Varanini, Tiziana Pandolfini

**Affiliations:** ^1^Faculty of Science and Technology, Free University of Bozen-BolzanoBolzano, Italy; ^2^Department of Biotechnology, University of VeronaVerona, Italy

**Keywords:** grape, wine, ionomic profile, traceability, rare earth elements, ICP-MS

## Abstract

The determination of food geographical origin has been an important subject of study over the past decade, with an increasing number of analytical techniques being developed to determine the provenance of agricultural products. Agricultural soils can differ for the composition and the relative quantities of mineral nutrients and trace elements whose bioavailability depends on soil properties. Therefore, the ionome of fruits, vegetables and derived products can reflect the mineral composition of the growth substrate. Multi-elemental analysis has been successfully applied to trace the provenance of wines from different countries or different wine-producing regions. However, winemaking process and environmental and cultural conditions may affect a geographical fingerprint. In this article, we discuss the possibility of applying ionomics in wines classification on a local scale and also by exploiting grape berry analyses. In this regard, we present the ionomic profile of grapevine berries grown within an area of approximately 300 km^2^ and the subsequent application of chemometric methods for the assignment of their geographical origin. The best discrimination was obtained by using a dataset composed only of rare earth elements. Considering the experiences reported in the literature and our results, we concluded that sample representativeness and the application of a preliminary Principal Component Analysis, as pattern recognition techniques, might represent two necessary starting points for the geographical determination of the geographical origin of grape berries; therefore, on the basis of these observations we also include some recommendations to be considered for future application of these techniques for grape and wines classification.

## Introduction

The geographical origin and the authenticity of food products are often related to the overall perception that consumers have in terms of quality, thus having a strong impact on the commercial value of the goods. In the last decades, fingerprinting techniques based on the chemical analyses of agricultural products followed by multivariate statistical approaches have been developed, aiming at identifying and classifying products according to their geographical origin (Versari et al., 2014). These methods assume that the chemical composition of the food product under study (e.g., mineral elements, stable isotopes ratios, and metabolites) is depending on the provenance environment (Versari et al., 2014). The fingerprinting methods based on mineral element composition of food stuff have been largely adopted in the last years to trace the geographical origin of wine, olive oil, honey, cheese, coffee, vegetable, fruits, and spices (Danezis et al., 2016). One of the most popular techniques adopted for these analyses is the inductively-coupled plasma mass spectrometry (ICP-MS), which can be used for the determination of both the ionomic profile and the isotope ratios (Baxter et al., 1997; Rebolo et al., 2000; Wieser et al., 2001; Castiñeira et al., 2004; Coetzee et al., 2005; Šelih et al., 2014; Capici et al., 2015; Mimmo et al., 2015; Popescu et al., 2015; Scampicchio et al., 2016).

In the particular case of agricultural products, it is postulated that the presence and the concentration of the mineral elements might reflect their geographical provenance (Almeida and Vasconcelos, 2003). Considering that the natural diffusion of mineral elements follows a pathway starting from the rocks, going through the soil and, finally, reaching the plant, it is thus conceivable that ionomic profile of plant organs and tissues is dependent on the geochemistry of the soil on which crops are cultivated (Geana et al., 2013). In addition, also anthropogenic activities, including the soil management, the use of fertilizers and phytochemicals, might determine alterations in the ionomic signature of agricultural products (Pepi et al., 2016a).

## Traceability of Wines

Among a wide variety of experiences dedicated to the geographical tracing of food products, a large number of studies, aiming at finding out reliable fingerprinting methods, have been carried out on wines traceability, most probably due to their relatively high commercial value. For these reasons, the elemental composition of different type of wines have been investigated with the aim of correlating them to the provenance soil for geographical tracing purposes (for an extensive review see Versari et al., 2014) (**Table 1**). However, the critical reading of the scientific literature published in this field of research demonstrates that the determination of the chemical descriptors for the origin of wines are strongly dependent on a plethora of factors, as for instance the number of samples used in the analyses, the type of wine (i.e., white, red, or rosè), the pattern recognition technique applied for the statistical analysis [e.g., Discriminant Analysis, Principal Component Analysis (PCA), Cluster Analysis, Stepwise Linear Discriminant Analysis and similar] and, most importantly, the geographical origin (Baxter et al., 1997; Díaz et al., 2003; Marengo and Aceto, 2003; Castiñeira et al., 2004; Jos et al., 2004; Thiel et al., 2004; Coetzee et al., 2005, 2014; Angus et al., 2006; Capron et al., 2007; Galgano et al., 2008; Serapinas et al., 2008; Forina et al., 2009; Fabani et al., 2010; Catarino et al., 2011; Rodrigues et al., 2011; Martin et al., 2012; Zou et al., 2012; Azcarate et al., 2013; Geana et al., 2013; Šelih et al., 2014). As also shown in **Table 1**, the majority of geographical tracing studies explores the analytical dataset by means of unsupervised pattern recognition analyses (e.g., PCA) and, once the most discriminant variables have been found, *ad hoc* statistical analyses, specifically supervised methods, are run in order to exacerbate the clusterization and to extract further information from the dataset. In addition, it could also be inferred that the power of the technique exploited for the chemical analysis (i.e., AAS, ICP-OES, ICP-AES, and ICP-MS) might be determinant for the tracing purposes.

**Table 1:** Recent examples of applications of ionomic analysis to wine classification according to their geographical origin.

Type of samples included in the analysis	Number of samples analyzed	Country	Number of geographical region considered	Number and type of elements used for the classification	Data analyses	Reference
Wine	40	South Africa	3	12 Al, Ba, Cs, Ga, Mn, Ni, Rb, Sc, Se, Sr, Tl, W.	PCA	Coetzee et al., 2005
Wine Soil	31 wines 137 soil samples	Argentina	3	7 K, Fe, Ca, Cr, Mg, Zn, Mn.	LDA	Fabani et al., 2010
Wine Soil Must	4	Portugal	3	14 La, Ce, Pr, Nd, Sm, Eu, Gd, Tb, Dy, Ho, Er, Tm, Yb, Lu.	Distribution patterns	Catarino et al., 2011
Wine Soil	60 wines 19 soil samples	Romania	3	12 Ni, Ag, Cr, Sr, Zn, Cu, Rb, Mn, Pb, Co, V, Be	PCA	Geana et al., 2013
Wine	120 wines	South Africa	23 estates in a region of 1000 km^2^	9 B, Ba, Cs, Cu, Mg, Rb, Sr, Tl, Zn.	PCA CA DA	Coetzee et al., 2014
Wine	185	Slovenia	4	19 Al, Ba, Ca, Cu, Fe, K, Mg, Mn, Na, Zn, Cr.	PCA CPANN	Šelih et al., 2014
Wine	57	Argentina	4	5 Ba, As, Pb, Mo, Co.	PCA LDA	Azcarate et al., 2013


*CA, cluster analysis; CPANN, counter-propagation artificial neural networks; DA, discriminant analysis; LDA, linear discriminant analysis; PCA, principal component analysis.*

Therefore, the use of multi-elemental profile of wines as a fingerprinting technique requires the careful identification of suitable elements that generally reflect the characteristic features of the provenance soil. Thus, the correlation between chemical composition of wine and provenance soil is usually considered an important prerequisite for classification of wines according to geographical origin. In wine analyses, the choice of the indicator elements should also take into account possible distortions due to agricultural practices, environmental conditions and winemaking process.

Winemaking is a complex process that involves multiple stages, as for instance blending, fermentation, rectification, and clarification, which have shown to influence element concentration in the final product. Early studies have shown that the concentration of elements may either increase (e.g., Al, Cd, Cr, Fe, Pb, and V) (Kristl et al., 2002; Almeida and Vasconcelos, 2003) or decrease (i.e., Al, Cd, Co, Cr, Fe, Pb, and V) (Eschnauer et al., 1989) in the processes of must fermentation and wine fining. More recently, Aceto et al. (2013) carried out a geographical tracing study on Moscato wines and demonstrated that the concentration of lanthanides, used as chemical markers, is conserved from soil to must, whilst the fingerprinting was affected by the treatments with bentonites. These observations led to the conclusion that wine traceability could be only pursued if the fining treatments were alternative to the bentonites ones (Aceto et al., 2013).

## Geographical Origin of Grape Berries Using Rare Earth Elements As Chemical Descriptors: The Verona Region Case

Some of the limitations in wine fingerprinting, as discussed above, may be overcome by analyzing the chemical composition of berries. In particular, this would circumvent the problems associated with the chemical changes caused by winemaking, in particular for Rare Earth Elements (REEs).

It is widely accepted that the composition in terms of REEs in the rocks is reflected also in the soil and in the plant tissues, even though a certain degree of variability is observed depending on the plant species (Wang et al., 1997; Wyttenbach et al., 1998; Zhang et al., 2002; Oddone et al., 2009). In the case of *Vitis vinifera*, the distribution of REEs within the berries has been studied in different cultivars (e.g., Chardonnay, Cabernet Sauvignon, Italian Riesling) by ICP-MS techniques (Bertoldi et al., 2009; Yang et al., 2010). In particular, Bertoldi et al. (2009) were able to show that Europium was accumulated in grape berries seed. Collectively, the results obtained within these studies prompted other authors to exploit these features (i.e., REEs) to study the geographical origin of wines, also considering the recent evidence demonstrating that different rootstocks do not significantly affect the REE content in the grape berries (Pisciotta et al., 2017).

For instance, the afore-mentioned Moscato tracing work represents a comprehensive study in which the wine production chain has been investigated for geographical discrimination purposes and the correlation between the soil composition in terms of REEs and their concentration of berries and musts has been examined (Aceto et al., 2013). The unsupervised pattern recognition analyses carried out on musts did not highlight any difference between samples collected in the Moscato DOCG geographical region (Aceto et al., 2013). On one hand, these results indeed highlighted the power of REEs to assess the belonging of Moscato samples to the DOCG area; on the other, no striking distinctions between the different vineyards were found. Further insight in the use of REEs for the determination of geographical provenance was obtained with a study considering the REEs profile in berries of the “Glera” cultivar sampled in five different vineyards in the Veneto region, Italy (Pepi et al., 2016b). The authors established a correlation between the REEs concentration in the berries and REEs available fraction in the soil; this indeed allowed the discrimination of the provenance (Pepi et al., 2016b), provided that the geological origin of the soil in the vineyard considered across the Veneto region was fairly diverse.

Few examples also indicated the possibility to use the trace element composition for classification of wines produced in wine growing regions located in small geographical area (Coetzee et al., 2005; Šelih et al., 2014). In this context, we decided to investigate whether the ICP-MS multi-elemental analysis followed by multivariate statistical analyses could be effective in the distinction of grape samples originating from neighboring vineyards (within an area of 300 km^2^). We harvested berries of *V. vinifera* cv. Corvina (clone 48) at full ripening stage (Brix degree ranging between 18 and 24) from eleven vineyards located in the three most important wine production macro-areas of the Verona region, namely Bardolino, Valpolicella, and Soave. The sampling rationale and procedures, as well as the sampling sites, were previously described by Dal Santo et al. (2013) and Anesi et al. (2015). The samples were homogenized, mineralized, and the concentration of 34 mineral elements in grapevine berries was determined by ICP-MS. These data were used as chemical descriptors to establish, through chemometric methods, criteria for assigning their geographical origin.

**FIGURE 1:**
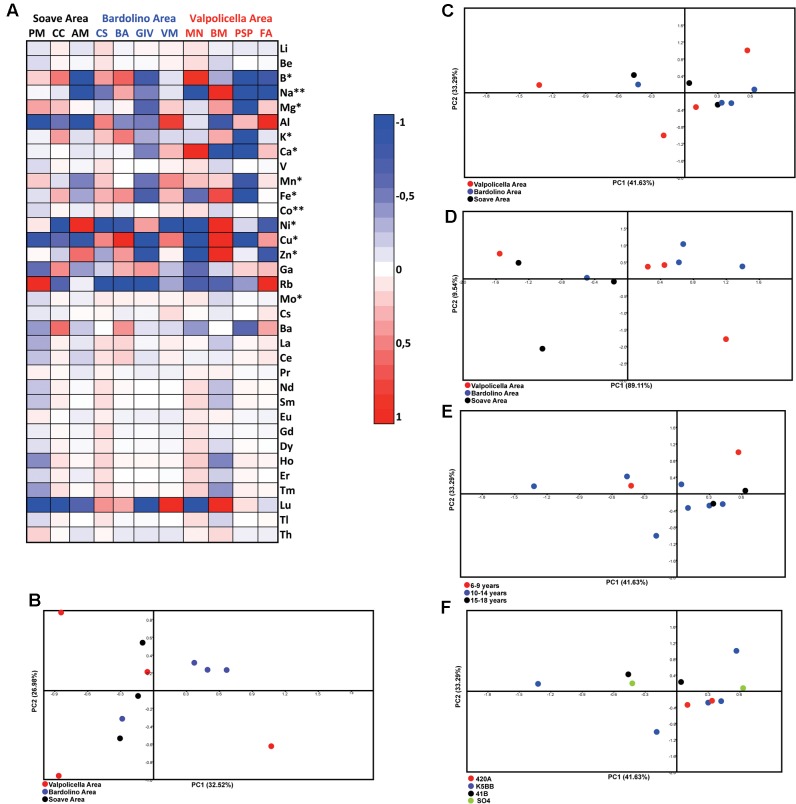
**(A)** Heat map reporting the variation of the mineral elements concentration in the samples considered in the analyses. ^∗^ indicates essential nutrients; ^∗∗^ indicates beneficial nutrients. **(B)** Principal components analysis. The scatterplot has been obtained analyzing the whole dataset obtained through the multi elemental ICP-MS analysis. The model generated by PCA analysis is formed by six components accounted for 97.66% of the total variance. The PC1 and the PC2 used for the scatter plot accounts for 59.50% of the total variance. **(C)** The PCA analysis was carried out considering only micro-, macro- and beneficial elements generating a model composed of five components, which described 96.97% of the total variance. The scatter plot was obtained by combining the first two components, accounting for 74.92% of the variance. **(D)** The PCA analysis was carried out considering only REEs generating a model composed of two components, which describe 98.65% of the total variance. **(E,F)** Scatter plot obtained by PCA of the sub-data set encompassing only micro-, macro- and beneficial elements. The age of the vineyards **(E)** and the rootstock genotype **(F)** are highlighted.

In order to obtain a comprehensive view of the whole dataset, the concentration of mineral elements was used to build a heat map, in which each value has been calculated as the log2 of the ratio between the element concentration in the sample and the average concentration of that element in all the samples (**Figure 1A**). Within the heat map, it was possible to differentiate two big groups of elements: a first group, encompassing mostly plant macro- and micronutrients (except for Ga and Rb), which showed strong variations, both positive and negative, and a second group that was formed mostly by REEs, which showed milder fluctuations between samples (**Figure 1A**). Micro- and macronutrients, as well as beneficial elements (Marschner, 2011), are actively taken up, accumulated, and differentially allocated in tissues and organs, and their homeostasis is tightly regulated in order to avoid nutritional imbalances (Williams and Salt, 2009). Therefore, substantial variations in the micro- and macronutrient concentrations are to be expected, depending on the soil type and on the rootstock genotype. On the other hand, REEs are not essential to plants; still they can be absorbed following the route of Ca, with which they share a similar ionic radius (Pickard, 1970; Hu et al., 2004). This is supported by the observations that Ca can be replaced by REEs in several biochemical and physiological functions (Pickard, 1970; Hu et al., 2004; Liu and Hasenstein, 2005; Babula et al., 2008; Xiaoqing et al., 2009; Carpenter et al., 2015; Yang et al., 2015). Besides the natural variations of element concentrations due to soil characteristics and origin, it is noteworthy that Cu, in 5 samples out of 11, was more abundant as compared to the global average value (**Figure 1A**). This behavior, also documented by Geana et al. (2013) in Romanian wine samples, might be due to Cu accumulation in soil, following the agronomical practice of using Cu-based fungicides for the protection of grapevine plants against downy mildew.

Pattern recognition analyses were carried out on the whole dataset in order to highlight possible differences and similarities among the samples considered, finally aiming at the geographical origin discrimination. The PCA generated a six-component model, accounting for a total variance of 97.66%. The first two components, which together explained about 59.50% of the total variance, have been used to graphically represent the model (**Figure 1B**). The validity of the PCA models were assessed by the cross-validation approach previously described Bro et al. (2008) and Pii et al. (2015). Despite accounting for more than half of the total variance, the model obtained failed in describing the geographical provenance of samples, since they resulted randomly scattered across the diagram, except for three samples belonging to the Bardolino area that closely clustered together in the same quarter (**Figure 1B**).

According to differences in element behavior displayed in the concentration heat map (**Figure 1A**), the whole dataset was split into two sub-datasets, the first encompassing micro-, macro- and beneficial elements, and the second comprising REEs, and they were subjected to PCA (**Figures 1C,D**).

The multivariate analysis of the first sub-dataset (i.e., micro-, macro- and beneficial elements) generated a five components model, accounting on the whole for 96.96% of the total variance. The scatter plot obtained combining the first two components, which represented 74.92% of the variance, showed neither the separation of samples according with the geographical origin nor any other clear clustering (**Figure 1C**). Indeed, the distribution along the first component was mainly driven by Cu and, to a lower extent, by Fe, Na and B (Data not shown). As previously discussed, the differential accumulation of Cu could be due to agronomical practices (Geana et al., 2013); nonetheless, it has also been observed that the element composition of berries is also dependent on the rootstock genotype (Gąstoł and Domagała-Świtkiewicz, 2013). For this reason, both the age of the vineyard and the rootstock genotype have been highlighted within the PCA model discussed above (**Figures 1C,E,F**). In spite of this, any clear clustering regarding the classification of the samples considered (i.e., vineyard age and rootstock genotype) was obtained (**Figures 1E,F**).

On the other hand, when only the REEs were considered for PCA, a two-components model accounting for 98.65% of the total variance has been obtained (**Figure 1D**). The scatterplot showed the separation of samples into two distinct clusters along the first component of the model, one encompassing samples from Bardolino and Valpolicella vineyards and the other comprising Soave vineyards. Nevertheless, two outliers from the other vineyards clustered with Soave samples (**Figure 1D**). According to the loading plot, the separation along the first component was mainly driven by Lu, whereas the other REEs contributed to the separation of samples along the second component (data not shown). This behavior might be due to the fact that Lu showed the strongest variation in concentration among the REEs group (**Figure 1A**). To the best of our knowledge, Lu has not emerged as discriminant element yet.

## Conclusion and Recommendations for Future Studies

In conclusion, the new data presented here showed that the whole ionomic signature of the grape berries did not fully allow the discrimination of their geographical origin, most likely due to the heterogeneity in the characteristics (i.e., vineyards age, rootstock genotypes and agricultural practices) of the vineyards and the limited number of samples analyzed. Nonetheless, our data confirm that the multielemental analyses based on REEs of agricultural products might be a powerful technique to trace the geographical origin of foodstuff, and, in this specific case, of grapevine berries and musts. Furthermore, our data indicate that the ionomic signature can be suitable even for agricultural products originating from neighboring regions.

On the bases of this experience and the pieces of research published in the literature, we suggest making the following recommendations, which may be considered in the experimental design, aiming at improving the efficacy and the resolution of the predictive tool:

(1) A preliminary analysis of vineyard soil could be useful for the “early” identification of chemical markers.
(2) The analysis of both berry and wine samples can be successfully used for the accurate classification at both regional and sub-regional levels.
(3) REE are powerful markers for berries analyses since these elements are not greatly affected by external interferences such as agricultural practices or environmental conditions and show a very little dependence on the grapevine rootstock. However, they can be unsuitable for wine analyses since winemaking processes can affect their concentrations.
(4) The number of samples to be included in the study should be determined at the beginning, according to the desired precision of the model, even though no clear indications can be deduced from the literature, since highly variable sample sizes have been used for such studies. However, a balanced number of samples and their representativeness might be a critical feature; in this regard, the maximization of the variability that describes a vineyard (i.e., cultivation practices, microclimatic conditions and altitude) could be of paramount importance to obtain a robust predictive model.
(5) The application of a single statistical analysis might result in a reduced extraction of information from the dataset. In general, unsupervised methods, as PCA, should be applied for the initial exploration of the dataset and to highlight the most discriminant features; next, supervised classification approaches can be run to refine the clusterization and to capture further information hidden in the dataset.

The commercial value of wines greatly depends on the authentication of their geographical origin, which represents a benefit for both consumers and wine producers. The ionomic signature appears as a powerful and flexible method to trace wine provenance even at the level of wine-producing sub-regions. Its flexibility relies on the availability of multiple elemental markers, different types of samples (wine, must, grape) for chemical analysis and numerous analytical and statistical methods. The optimization of these parameters, as well as the application of a sufficiently large number of variables, may allow tailoring the experimental set up for each wine-making area.

## Author Contributions

YP, AZ, ZV, and TP: Designed the experiments. YP and AZ: Samples and Data Analyses. SD and MP: Provided the samples and information about the vineyards. YP, AZ, ZV, and TP: Critical discussion of the data. YP and TP: Paper preparation. TP: Research coordination.

## Conflict of Interest Statement

The authors declare that the research was conducted in the absence of any commercial or financial relationships that could be construed as a potential conflict of interest.
